# Supplementary material for: Genotypic and phenotypic characterization of Enterococcus faecalis isolates from periprosthetic joint infections
Source: Microbiol Spectr. 2024 Jun 24;12(8):e00565-24. doi: 10.1128/spectrum.00565-24 (PMC11302728; doi:10.1128/spectrum.00565-24)
Supplement: Supplemental material — Tables S1 and S2; Fig. S1 to S7. [file spectrum.00565-24-s0001.docx]

**Supplementary Table 1. Plasmid information.**

| **Strain** |  | **Found in** | |
| --- | --- | --- | --- |
|  | **Length (bp)** | **BV-BRC** | **PlasmidFinder** |
| **IDRL-7415** | 53,449 | yes | yes |
| **IDRL-7538** | 83,692 | yes | yes |
|  | 57,643 | yes | yes |
| **IDRL-9065** | 747,526 | yes | yes |
|  | 71,938 | yes | no |
|  | 71,205 | yes | yes |

**Supplementary Table 2: Antibiotic disk diffusion assay values.**

|  |  | |  |  | **OG1RF** | **IDRL-7415** | **IDRL-7538** | **IDRL-7639** | **IDRL-9065** |
| --- | --- | --- | --- | --- | --- | --- | --- | --- | --- |
|  | **Ampicillin** | | +SSF | Mean (mm) | 29.76 | 25.62 | 34.01 | 30.86 | 30.80 |
|  |  |  |  | SD | 3.80 | 6.57 | 3.02 | 6.34 | 3.57 |
|  |  |  | -SSF | Mean (mm) | 29.97 | 29.88 | 32.10 | 28.42 | 32.24 |
|  |  |  |  | SD | 2.96 | 2.22 | 4.37 | 1.46 | 1.93 |
|  | **Penicillin** | | +SSF | Mean (mm) | 24.19 | 23.29 | 21.40 | 24.76 | 12.98 |
|  |  |  |  | SD | 3.33 | 2.92 | 13.34 | 5.43 | 12.09 |
|  |  |  | -SSF | Mean (mm) | 25.11 | 23.72 | 26.38 | 23.31 | 26.36 |
|  |  |  |  | SD | 2.25 | 1.01 | 1.97 | 1.93 | 1.25 |
|  | **Cephalothin** | | +SSF | Mean (mm) | 6.00 | 6.00 | 6.00 | 6.00 | 6.00 |
|  |  |  |  | SD | 0.00 | 0.00 | 0.00 | 0.00 | 0.00 |
|  |  |  | -SSF | Mean (mm) | 16.83 | 6.00 | 6.00 | 6.00 | 7.38 |
|  |  |  |  | SD | 9.40 | 0.00 | 0.00 | 0.00 | 2.38 |
|  | **Ceftriaxone** | | +SSF | Mean (mm) | 22.76 | 18.74 | 20.10 | 19.54 | 10.90 |
|  |  |  |  | SD | 4.37 | 3.86 | 8.72 | 4.00 | 8.49 |
|  |  |  | -SSF | Mean (mm) | 20.60 | 19.80 | 19.35 | 17.40 | 14.02 |
|  |  |  |  | SD | 2.27 | 1.29 | 1.70 | 2.14 | 6.96 |
|  | **Gentamicin** | | +SSF | Mean (mm) | 19.52 | 19.19 | 7.60 | 12.97 | 21.57 |
|  |  |  |  | SD | 6.05 | 2.67 | 2.77 | 6.41 | 6.72 |
|  |  |  | -SSF | Mean (mm) | 16.62 | 18.54 | 16.16 | 17.96 | 18.12 |
|  |  |  |  | SD | 1.20 | 0.81 | 2.72 | 2.56 | 0.34 |
|  | **Linezolid** | | +SSF | Mean (mm) | 27.94 | 30.03 | 29.11 | 30.27 | 32.18 |
|  |  |  |  | SD | 3.81 | 4.30 | 3.57 | 5.13 | 6.51 |
|  |  |  | -SSF | Mean (mm) | 26.61 | 27.77 | 27.91 | 29.61 | 28.89 |
|  |  |  |  | SD | 2.28 | 3.64 | 2.83 | 3.59 | 3.46 |
|  | **Minocycline** | | +SSF | Mean (mm) | 29.48 | 20.57 | 25.26 | 29.16 | 21.77 |
|  |  |  |  | SD | 4.03 | 1.47 | 3.08 | 9.15 | 7.70 |
|  |  |  | -SSF | Mean (mm) | 30.25 | 16.89 | 21.39 | 27.68 | 18.06 |
|  |  |  |  | SD | 3.40 | 1.91 | 4.04 | 6.81 | 1.60 |
|  | **Vancomycin** | | +SSF | Mean (mm) | 15.45 | 15.48 | 17.99 | 19.08 | 17.68 |
|  |  |  |  | SD | 1.93 | 3.77 | 3.09 | 6.11 | 1.91 |
|  |  |  | -SSF | Mean (mm) | 15.59 | 15.58 | 17.09 | 15.60 | 14.82 |
|  |  |  |  | SD | 1.42 | 1.99 | 1.21 | 1.07 | 1.30 |
|  |  |  |  |  |  |  |  |  |  |

**
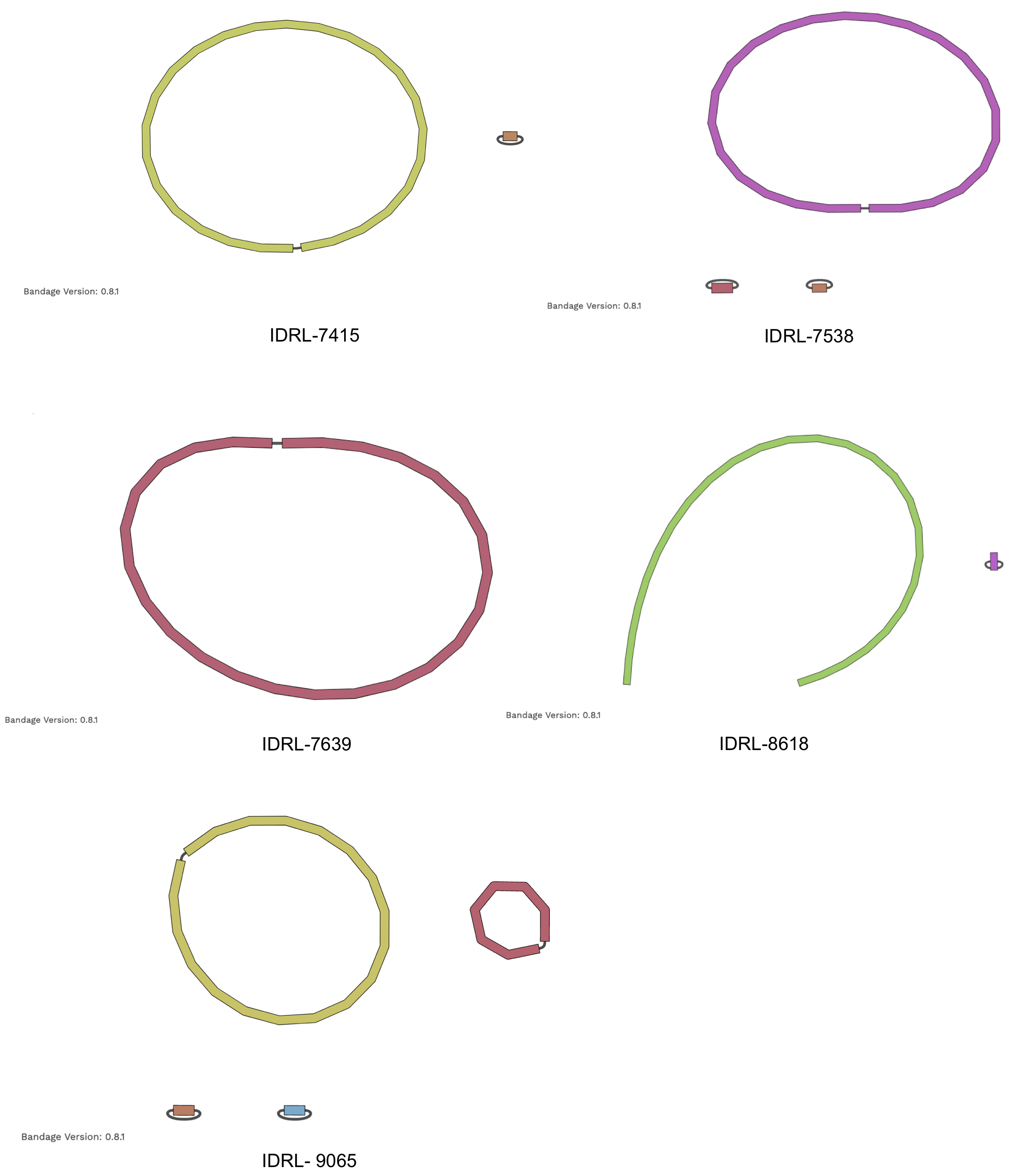
**

**Supplementary Figure 1. Bandage plots for assembled genomes of *E. faecalis* PJI isolates.** Bandage plots were generated using Unicycler to evaluate assembled genomes. Chromosomes are represented by large circles (or the large linear segment for IDRL-8618), and plasmids are represented by smaller circles.

**
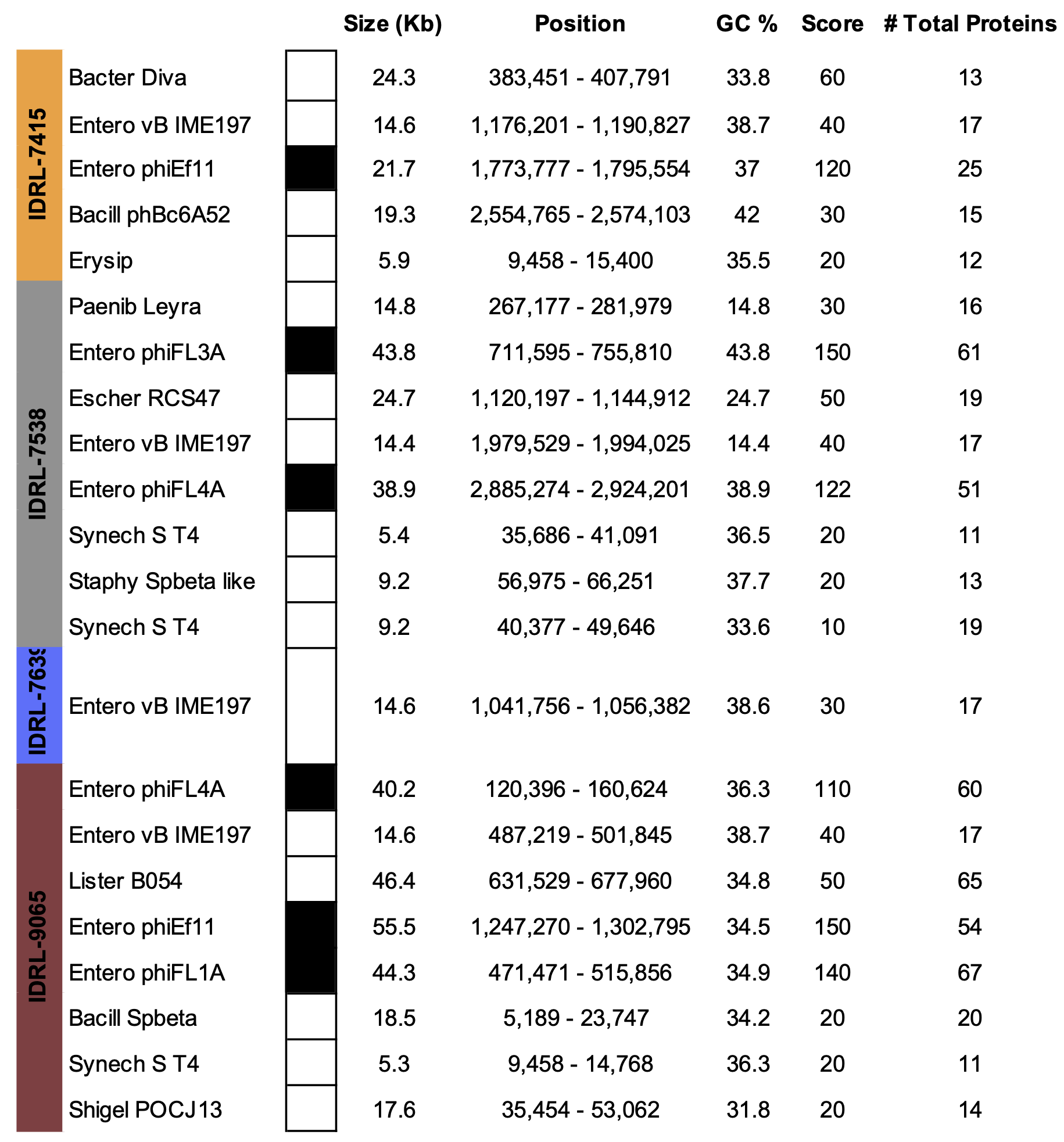
**

**Supplementary Figure 2: Predictions of prophages within complete *E. faecalis* PJI isolate genomes using Phaster.** Colored blocks indicate complete (black) or incomplete (white) prophages. The score column is based on region completeness and length and is used to predict intact (>90), questionable (70-90), or incomplete (<70) prophage regions.

**Supplementary Figure 3. Length of cell chains in *E. faecalis* biofilms.** The length of multicellular chains was quantified in biological replicates (n = 2 or 3) of biofilms grown in BHI. Statistical significance was determined using ordinary one-way ANOVA with Tukey’s multiple comparisons. ** = P ≤ 0.01, *** = P ≤ 0.001, **** = P ≤ 0.0001.

**
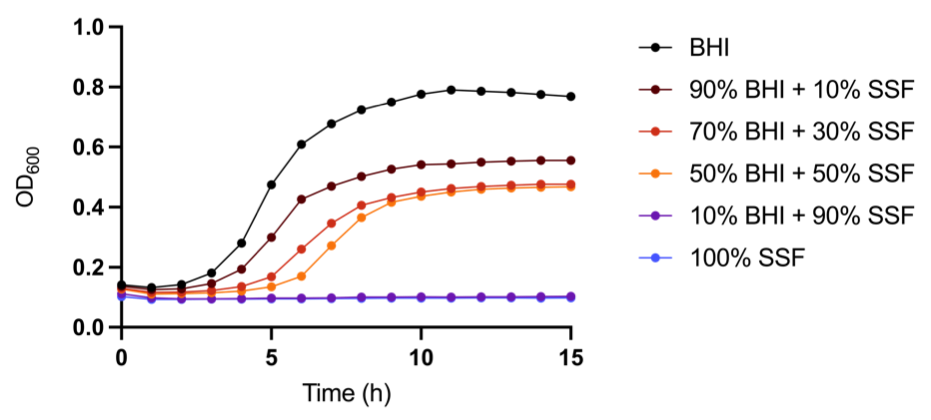
**

**Supplementary Figure 4. SSF growth optimization for OG1RF.** Growth of OG1RF in BHI supplemented with SSF. Data represents an average of three technical replicates from n = 2 independent biological replicates.

**
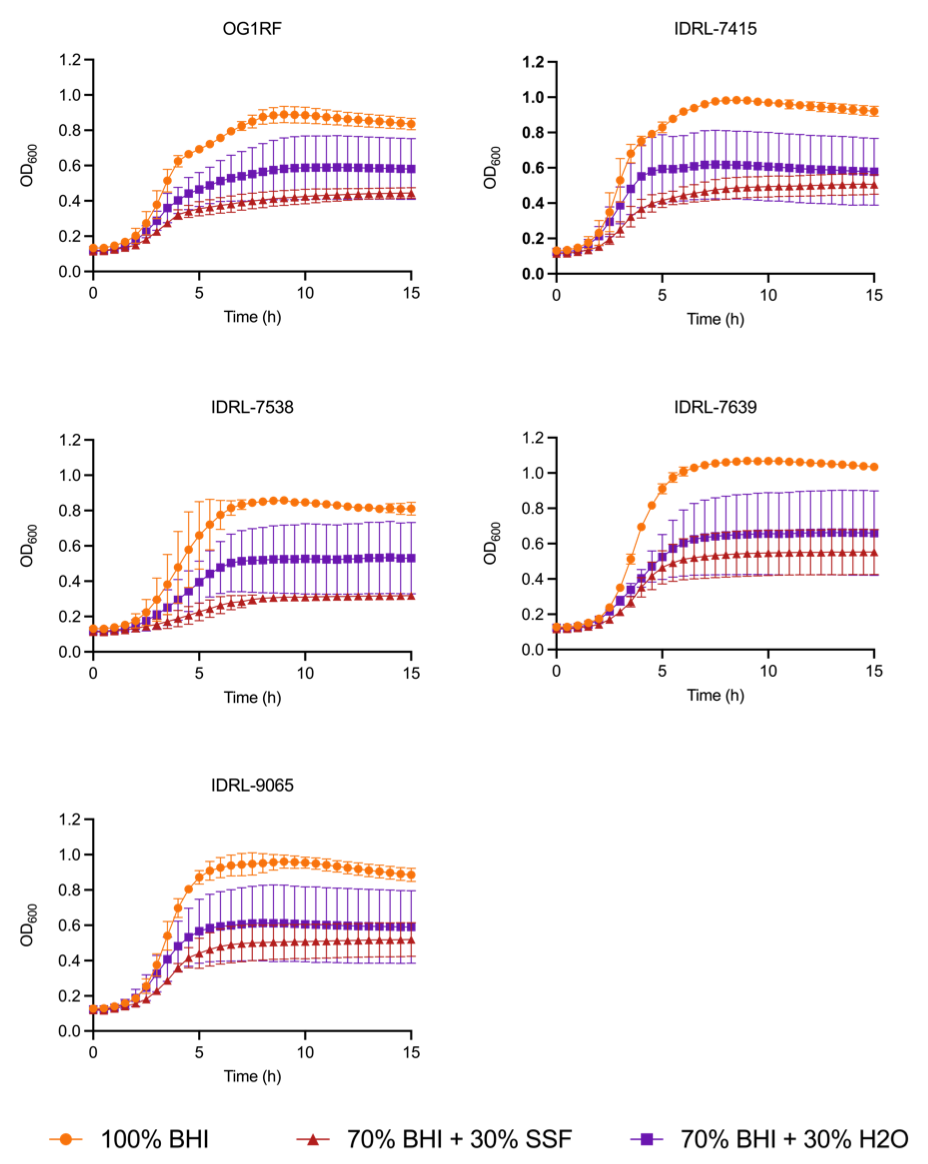
**

**Supplementary Figure 5:** Growth curves of each isolate in BHI and SSF. 15-hour growth curves of each clinical isolate with 100% BHI or 70% BHI + 30% SSF. Data represents a mean and SD for n=3 biological replicates, each averaged from triplicate technical replicates.

**

**

**Supplementary Figure 6. Individual OD_600_ and OD_450_ values for biofilm figures.** Individual values representing growth (OD_600_) and biofilm formation (OD_450_) for 96-well plate biofilm assays are shown. Each dot represents an individual biological replicate (average of three technical replicates). Media conditions are color-coded (gray, BHI; blue, 70% BHI/30% H_2_O; purple, 70% BHI/30% SSF). The error bars represent standard deviation. These raw values were used to calculate biofilm index (OD_450_/ OD_600_), which is shown in Figure 3 and Figure 4.

**
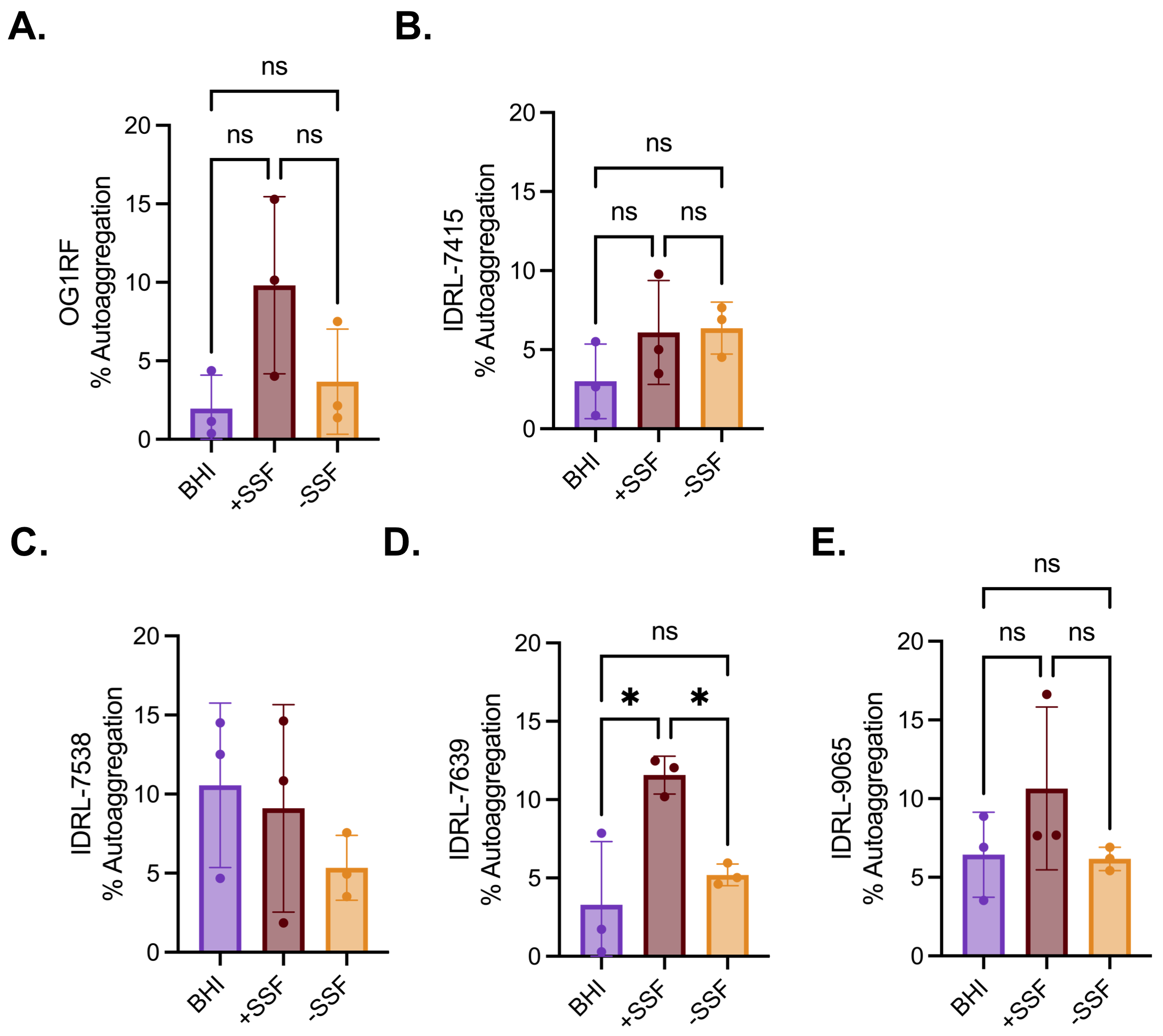
**

**Supplementary Figure 7. Autoaggregation of *E. faecalis* PJI isolates in shaking conditions.** Each clinical isolate was subject to growth in 100% BHI, 70% BHI + 30% SSF and 70% BHI + 30% H_2_O for 16-18 h with shaking at 37 ^o^C. Data represents n = 3 independent biological replicates. Statistical significance was determined using ordinary one-way ANOVA (*p<0.05).
